# Analysis of Root Canal Anatomy of Mandibular Permanent Incisors in Saudi Subpopulation: A Cone-Beam Computed Tomography (CBCT) Study

**DOI:** 10.1155/2022/3278943

**Published:** 2022-05-19

**Authors:** Amal Almohaimede, Alanoud Alqahtani, Norah Alhatlani, Nouf Alsaloom, Shafia Alqahtani

**Affiliations:** ^1^Department of Restorative Dental Sciences, Endodontic Division, College of Dentistry, King Saud University, P.O.Box: 5967, Riyadh 11432, Saudi Arabia; ^2^Saudi Board Endodontic Residency Program, King Faisal Specialist Hospital Research Center, Riyadh, Saudi Arabia; ^3^Saudi Board Pedodontics Residency Program, King Saud Medical City, Riyadh, Saudi Arabia; ^4^Saudi Board Periodontics Residency Program, Princess Norah Bint Abdulrahman University, Riyadh, Saudi Arabia; ^5^Saudi Board Orthodontics Residency Program, King Abdulaziz Medical City, Riyadh, Saudi Arabia

## Abstract

This study aimed to evaluate the root canal anatomy of central and lateral mandibular incisors in a Saudi population using cone-beam computed tomography (CBCT). Overall, 1370 CBCT images of central (687) and lateral (683) mandibular incisors of Saudi patients who attended the Dental College at King Saud University in Riyadh were examined. The number of roots and canals, canal configuration types, symmetry between bilateral incisors, and the effect of gender and age were determined. For data analysis, the chi-square test was applied, and the *p* value was set at ≤0.05. Only one tooth had two roots, and 41% of mandibular incisors had two canals. The most common canal configuration type observed was type I (58.83%), followed by type III (28.24%). Type V was more common in men (8.31%) than women (3.9%). Bilateral symmetries were higher in the mandibular central incisors regarding the root and canal numbers and the canal configuration types (100, 100, and 97.92%, respectively) than in the lateral incisors (99.69, 98.16, and 97.24%, respectively). The 21–40 age group showed a higher proportion of teeth with more complicated root canal anatomy than the other age groups. More than one canal in mandibular incisors is a common finding in the Saudi subpopulation, with the type III canal configuration as the most common type.

## 1. Introduction

Successful root canal treatment requires sufficient awareness of tooth morphology [1]. Inability to locate root canals may lead to endodontic treatment failure [2]. Von Arx found that the presence of isthmuses and untreated canals are the main causes of endodontic therapy failure [3]. Baruwa et al. documented the incidence of untreated root canals in lower incisors at 29.6% [4]. Several investigations have evaluated the root canal anatomy of the lower incisors. Vertucci examined 200 lower central and lateral incisors using demineralization and dye injection and reported that 70% of central incisors and 75% of lateral incisors had a single canal with one foramen [5]. Green reported that 79% of the examined 500 mandibular incisors, using the grinding and staining technique, had one major canal with one apical foramen [6]. However, Sert et al. evaluated 400 mandibular incisors using the decalcification and staining technique and reported that 67.5% of central incisors and 63% of lateral incisors had more than a single canal [7]. Cone-beam computed tomography (CBCT) has been widely utilized in endodontic practice. It is used for the diagnosis and detection of apical periodontitis [8], the assessment of tooth morphology and additional roots and canals [9–11], presurgical assessment of the anatomical relationship of the root apices to important anatomical structures [12], and the assessment of traumatic injuries and sequelae [13]. Several studies have observed the root and canal anatomy of lower central and lateral incisors using CBCT as an evaluation tool [14–19]. The existence of a second root canal in mandibular central and lateral incisors using CBCT as an evaluation method ranged from 2.7% [19] to 44% [18]. In Saudi Arabia, five studies evaluated the root canal anatomy of central and lateral mandibular incisors in different regions [20–24]. Only one of them investigated the effect of age on the root canal anatomy of mandibular central and lateral incisors [23].

This study aimed to investigate the root canal morphology of mandibular central and lateral incisors, the differences between genders, age groups, and the bilateral symmetries in a Saudi subpopulation using CBCT.

## 2. Materials and Methods

This research was approved by the ethics committee at King Saud University (IRB Project No. E-17-2742). Overall, 1370 CBCT images of mandibular permanent incisors (central and lateral) of Saudi patients (577 men and 793 women) who visited the Radiology Department at Dental College at King Saud University in Riyadh between 2015 and 2019 were retrieved. These patients were aged between 18 and 74 years. Images of their lower permanent central and lateral incisors with complete root formation were included. Images of low quality, teeth with root resorption or periapical radiolucencies, previously treated or initiated teeth, teeth with immature apices, and the presence of coronal or postrestorations were excluded. Teeth were investigated for the number of roots and canals and canal configuration types according to Vertucci's classification [5,25]:

- Type I: A single canal extends from the pulp chamber to the apex.
- Type II: Two separate canals leave the pulp chamber then join to exit as one canal.
- Type III: One canal leaves the pulp chamber, divides into two within the root, and then joins to exit as one canal.
- Type IV: Two separate canals leave the pulp chamber and exit as two distinct canals.
- Type V: One canal leaves the pulp chamber and divides within the body of the root to exit as two separate canals.
- Type VI: Two separate canals leave the pulp chamber, join within the body of the root, and then redivide to exit as two distinct canals.
- Type VII: One canal leaves the pulp chamber, then divides and rejoins within the body of the root canal, and then redivides to exist as two separate canals.
- Type VIII: Three separate canals leave the pulp chamber and exit as three distinct canals.

Furthermore, the root, canal, and canal configuration symmetry between the bilateral incisors (centrals and laterals) were determined. Gender and age were recorded.

The CBCT images were interpreted at the Radiology Department of the Dental College at King Saud University by one endodontist and three trained interns for the number of roots and canals and types of canal configuration. Consultations were undertaken with an oral radiologist [26]. For image assessment, Planmeca Romexis Viewer software was utilized (Planmeca, Roselle IL). A professional technician acquired the radiological images according to the manufacturer's recommendation using Planmeca ProMax 3D (PLANMECA, Roselle, IL, USA) and a CS9300 3D digital imaging system (Carestream, Rochester, NY). The voxel size was 75–600 *μ*m, with small or large field of view (FOVs), the slice thickness was 0.2 mm viewed from the coronal to apical region, and the exposure time was 3–15 seconds [26].

To assess the intra- and interexaminer reliabilities, 20 CBCT images were arbitrarily chosen according to the evaluation criteria. Images were evaluated for tooth type (central or lateral), tooth side, number of roots and canals, and canal configuration type. Interexaminer agreement was assessed among the four different evaluators. To identify the intraexaminer agreement, the same images were reevaluated after one week by the same examiner.

### 2.1. Statistical Analysis

The Kappa test was applied for inter- and intraexaminer reliabilities [27]. The chi-square test was applied using SPSS 22 software (SPSS Inc., Chicago, IL) for data analysis. A *p* value ≤0.05 indicated statistical significance.

## 3. Results

All evaluators had kappa test values of 1 regarding the number of roots and canals. Regarding canal configuration type, the values were 1 for the first and fourth evaluators, 0.85 for the second evaluator, and 0.95 for the third evaluator. For the interexaminer reliability, almost perfect agreement (kappa test values = 1) between evaluators was observed for the number of roots and canals, and substantial agreement (kappa test value was 0.8) was observed for the root canal configuration. These values confirmed the reliability of the analysis performed by the evaluators.

The prevalence of teeth among different genders, tooth positions, and age groups is summarized in Table 1.

One tooth was recorded with two roots (0.1%); the mandibular right lateral incisor of a male patient. One root was recorded in 100% of lower central incisors and in 99.9% of lower lateral incisors; there were no significant differences between genders (*P*=0.421). The number of lower incisors reported with two canals was 564 (41.2%), and the remaining incisors had one canal 806 (58.8%), with no statistically significant difference noted between mandibular central and lateral incisors (*P*=0.38) [Table 2]. Although women had a higher number of mandibular incisors with two canals (322/57.09%) than men (242/42.9%), there was no statistically significant difference found (*P*=0.33).

**Figure 1:**
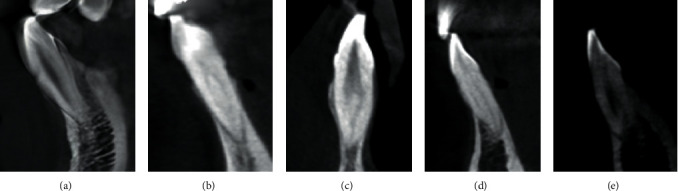
Sagittal CBCT sections of mandibular incisors show (a) one canal with type I Vertucci; (b) two canals with type II Vertucci; (c) two canals with type III Vertucci; (d) two canals with type IV Vertucci; (e) two canals with type V Vertucci.

The most frequent canal configuration type in mandibular incisors, present in 806 (58.83%), was type I, followed by type III in 387 (28.24%), type II in 88 (6.4%), type V in 79 (5.76%), and type IV in 10 (0.72%) Figure 1. No significant difference was found between the genders among the different types of canal configurations (*P* > 0.05), except type V that was more common in men than in women, with a statistically significant difference (*P*=0.008) Table 3.

No significant difference was found between the mandibular central and lateral incisors among the different types of canal configurations (*P* > 0.05), except type V was more common in mandibular lateral incisors than in mandibular central incisors, with a statistically significant difference (*P* ≤ 0.05) (Table 4).

### 3.1. Symmetry

Both mandibular left (337) and right (337) central incisors were found in 337 patients; 100% of central incisors showed symmetrical root and canal numbers, and 97.92% of teeth showed symmetrical canal configuration. Regarding mandibular lateral incisors, left (327) and right (327) teeth were found in 327 patients; 99.69% of lateral incisors had symmetrical root numbers, 98.16% had symmetrical canal numbers, and 97.24% had symmetrical canal configurations.

### 3.2. Age

Regarding age, a statistically significant difference was observed among the different age groups and the number of canals and the type of canal configuration (*P* ≤ 0.05). The 21–40 age group included a larger proportion of teeth with either one or two canals and a more complicated root canal anatomy than the other age groups [Table 5].

## 4. Discussion

This research investigated the morphological alterations in the root canal system of human permanent central and lateral mandibular incisors in a Saudi subpopulation using CBCT. Several studies previously documented the anatomy of mandibular incisors using different analysis techniques, such as staining and grinding [6], demineralization and staining [7,20,25], and, more recently, CBCT [21, 22, 28].

CBCT provides a three-dimensional examination of the anatomical structures. It has been documented as a valuable device to analyze root canal morphology [29, 30]. Moreover, its efficiency in evaluating the root canal system has been investigated. It was found that CBCT is comparable to the clearing and staining methods in its accuracy in detecting the number of root canals [31] or even superior to the clearing technique in identifying the type I Vertucci classification [32]. CBCT is considered a simple, practical, noninvasive, and reliable tool for evaluating root canal morphology [10, 33].

This investigation evaluated the root canal morphology of 687 lower central incisors and 683 lower lateral incisors using CBCT images. We found 100% of mandibular central incisors had a single root, 39.28% had two root canals, 99.9% of mandibular lateral incisors had a single root, and 43% had two root canals. Similar results regarding the number of roots but lower percentage of two canals were reported by Mashyakhy, who examined 410 lower central incisors and 412 lower lateral incisors in a Saudi Arabian population in the Jazan region using CBCT; he found that 100% of lower central incisors had a single root and 26.3% had two root canals versus 99.5% of lower lateral incisors with one root and 30.8% with two root canals [21]. In addition, Alkahtany et al. found similar results regarding the number of roots but lower percentage of two canals; they investigated the root canal morphology of 596 lower central incisors and 596 lower lateral incisors in a Saudi subpopulation using CBCT; all teeth had a single root and 22.31% of central incisors and 20.3% of lateral incisors had two canals [23]. Al-Fouzan et al. utilized the clearing technique to evaluate the root canal system of 80 extracted lower incisors (40 central incisors and 40 lateral incisors) obtained from Saudi patients; 30% of lower central incisors and 30% of lower lateral incisors had two canals [20], which was lower than the results found in this study. The same results were found by Mohamed et al. regarding the number of roots (single root in 100% of the examined teeth) after evaluating the root canal configuration of 188 lower central incisors and 188 lower lateral incisors among the Saudi subpopulation of the Qassim region; however, they reported lower results than ours regarding the presence of a second canal in the lower incisors (29.6% of lower central incisors and 26.1% of lower lateral incisors had two root canals) [24]. In Al-Madinah region in Saudi Arabia, Ghabbani et al. investigated 1624 mandibular incisors using CBCT. Similarly, all samples showed a single root, however, lower percentages of a second canal were recorded compared to ours results (25.02% of central incisors and 29.42% of lateral incisors had two root canals) [22]. The variations in the sample size and different regions may explain the difference in the results. The region of our study is considered a central region, the Jazan region is considered a southwestern region, and Al-Madinah region is considered as the western region.

Using CBCT, the incidence of a single-root canal in mandibular central and lateral incisors differed in different populations. In Germany, Baxter et al. reported that 24% of lower central incisors and 23.4% of lower lateral incisors had two root canals [14]. In Poland, Sroczyk et al. reported that 34.6% of lower central incisors and 32.8% of lower lateral incisors had two root canals [15]. In Iran, Mirhosseini et al. reported that 23.9% of lower central incisors and 35% of lower lateral incisors had two root canals [16]. In Malaysia, Pan et al. reported that 5.1% of lower central incisors and 12.2% of lower lateral incisors had two root canals [17]. In Italy, Valenti-Obino et al. reported that 45% of lower central incisors and 43% of lower lateral incisors had two root canals [18]. In China, Martins et al. reported that 0.4% of lower central incisors and 5% of lower lateral incisors had two root canals [19]. The same study evaluated the root canal anatomy of mandibular incisors in a Portugal population and reported that 27.4% of lower central incisors and 29.9% of lower lateral incisors had two root canals [19].

Five types of Vertucci root canal configurations were observed in the current study (types I, II, III, IV, V) [25]. The prevalence of having one apical foramen (types I, II, III) in mandibular central and lateral incisors was higher than that of two apical foramens (types IV and V): 96.1%, 90.92%, 3.9% and 9.1%, respectively. These results are consistent with the results of previous studies performed on the Saudi subpopulation by Al-Fouzan et al. [20], Mashyakhy [21], Ghabbani et al. [22], Alkahtany et al. [23], and Mohamed et al. [24]. Moreover, our results are consistent with the results of previous studies performed on different populations [14–19].

The present investigation examined bilateral symmetry in mandibular incisors. In mandibular central incisors, 100% of teeth showed an even number of roots and canals on both sides, and 97.92% of teeth showed symmetrical canal configuration on both sides. Regarding mandibular lateral incisors, 99.69% of teeth showed even numbers of roots and canals on both sides, 98.16% showed even canal numbers on both sides, and 97.24% showed even canal configurations on both sides. Our results are comparable to what was found by Mashyakhy in the Saudi subpopulation; he reported bilateral symmetry of the roots in 100% of central incisors and 99% of lateral incisors, while the prevalence of bilateral symmetry for canals were 91.2% and 85.8%, respectively [21]. Similarly, Ghabbani et al. reported that 98.8% of the examined left and right mandibular incisors in the Saudi subpopulation showed symmetrical root canal morphologies [22]. Likewise, using CBCT within a Turkish population, Kayaoglu et al. reported symmetries of the roots in 100% of lower central incisors and 99.8% of lower lateral incisors, and of the root canals in 94.8% lower central incisors and 89.8% lower lateral incisors [34]. Awareness about the presence of bilateral symmetry will aid the clinician during clinical practice in predicting the root canal anatomy on the contralateral side of the same patient.

Our study investigated the differences in the mandibular central and lateral incisor morphology by gender. No significant difference was found in terms of root and root canal numbers between the genders (*P* > 0.05). These results are in agreement with Mashyakhy's study of the Saudi population regarding lateral incisors. However, in central incisors, he found men had a significantly higher prevalence of two canals than women [21]. Likewise, in the Turkish subpopulation, it was reported that gender has no statistically significant correlation with the number of root canals [35]. Our results contradicted the results found by Alkahtany et al. in a Saudi population, who found that men had a significantly higher prevalence of two canals than women in mandibular incisors [23]. Moreover, in another Saudi population, Mohamed et al. found that women had a significantly higher prevalence of two root canals than men [24].

Studies on different populations regarding a correlation of gender with the number of root canals are contradictory. In an Indian subpopulation, the results showed that women had a significantly higher prevalence of two root canals than men [36]. On the other hand, in Caucasian [37] and Chinese populations [38], men had a higher prevalence of having more than one canal than women. These variations could be attributed to different sample sizes and different ethnic backgrounds.

The present study showed that a type V Vertucci's classification was more common in men than women, with a statistically significant difference (*P*=0.008). This result was in agreement with what was found by Basha in the Egyptian population [39]. On the other hand, Mashayakhy reported that in mandibular lateral incisors, there was no significant difference by gender among the different Vertucci canal classifications (type I, type III, and type V) [21].

This study also investigated the effect of age on root canal morphology. The findings showed that the 21–40 age group had a higher prevalence of teeth with two canals and more complicated root canal anatomy than the other age groups. Our results are consistent with Alkahtany et al., who found that the prevalence of two root canals was statistically higher in patients less than 40 years old [23]. Moreover, Karobari et al. evaluated root canal morphology in a Malaysian population and found that the 20–30 age group had more canal alterations in mandibular incisors [40]. Similarly, Kayaoglu et al., in their study in a Turkish subpopulation, found that patients over 56 years old had a lower frequency of two root canals [34]. Canal calcification and the deposition of secondary dentin are associated with increasing age [41]. Age-related modifications on dental pulp include the reduction of the pulp chamber caused by continuous formation of dentin [42], a reduced vascular supply, the formation of fibrous bundles, and the reduction of fibroblast density [43]. These changes start at 20–39 years of age followed by a decrease in odontoblast cellularity at 40–59 years of age [44]. These changes might explain the disappearance of extra root canals in older patients.

Our study had some limitations, such as the sample sizes among the three different age groups and the two genders were not equally distributed. Additionally, the study was limited to patients who attended Dental College at King Saud University. Moreover, the voxel size was massive in some cases (75–600 *μ*m) with large and small field of views (FOVs) which might lead to missing of some anatomy.

## 5. Conclusion

The presence of more than one canal in mandibular incisors is not uncommon. Therefore, comprehensive interpretation of radiographs, the extension of access preparation, and the use of dental microscopy to enhance visibility can help clinicians locate and negotiate extra canals.

## Figures and Tables

**Table 1:** The frequency of mandibular incisor teeth among different genders, tooth position, and age groups.

	Frequency of teeth (%)	Total
Gender:		
Male	577 (42.1%)	1370
Female	793 (57.9%)	
Tooth position:		
Mandibular left lateral incisor	338 (24.7%)	1370
Mandibular left central incisor	344 (25.1%)	
Mandibular right lateral incisor	345 (25.2%)	
Mandibular right central incisor	343 (25%)	
Age groups:		
18–20	351 (25.6%)	1370
21–40	617 (45.03%)	
>40	402 (29.34%)	

**Table 2:** The frequency of mandibular incisors teeth among different number of canals.

Frequency of teeth (%)		
Tooth type	With one canal (%)	With two canals (%)
Mandibular left lateral incisor	186 (13.57%)	152 (11.09%)
Mandibular left central incisor	208 (15.18%)	136 (9.92%)
Mandibular right lateral incisor	203 (14.81%)	142 (10.36%)
Mandibular right central incisor	209 (15.25%)	134 (9.7%)
Total	806 (58.8%)	564 (41.2%)

^*∗*^Significant at *P* ≤ 0.05.

**Table 3:** Variations in canal configuration types of mandibular incisor teeth among genders.

	Frequency of teeth (%)	
	Male (577)	Female (793)
Canal configuration type		
Type I	334 (57.88%)	472 (59.52%)
Type II	31 (5.37%)	57 (7.18%)
Type III	159 (27.55%)	228 (28.75%)
Type IV	5 (0.86%)	5 (0.63%)
Type V	48 (8.31%)^*∗*^	31 (3.9%)^*∗*^

^*∗*^Significant at *P* ≤ 0.05.

**Table 4:** The frequency of mandibular incisor teeth among different canal configuration types.

	Frequency of teeth (%)	
	Mandibular central incisors (687)	Mandibular lateralincisors (683)
Canal configuration type		
Type I	417 (60.69%)	389 (56.95%)
Type II	43 (6.25%)	45 (6.58%)
Type III	200 (29.11%)	187 (27.37%)
Type IV	6 (0.87%)	4 (0.58%)
Type V	21 (3.05%)^∗^	58 (8.49%)^∗^

^*∗*^Significant at *P* ≤ 0.05.

**Table 5:** The prevalence of canal number and different types of canal configurations among different age groups.

Age range groups (years old)	Prevalence of teeth (%)						
	Number of canals		Canal configurations				
	One canal	Two canals	Type I	Type II	Type III	Type IV	Type V
18–20	187 (13.64%)	164 (11.97%)	188 (13.72%)	38 (2.77%)	101 (7.37%)	9 (0.65%)	15 (1.09%)
21–40	367 (26.78%)^∗^	250 (18.24%)^*∗*^	367 (26.78%)^*∗*^	16 (1.16%)	187 (13.64%)^*∗*^	1 (0.072%)	46 (3.35%)^*∗*^
>40	252 (18.39%)	150 (10.94%)	251 (18.32%)	34 (2.48%)	99 (7.22%)	0	18 (1.31%)

^*∗*^Significant at *P* ≤ 0.05

## Data Availability

The data presented in this study are available on request from the corresponding author.
